# **Statistical** **Optimization of Oral Vancomycin-Eudragit RS Nanoparticles Using Response Surface Methodology**

**Published:** 2012

**Authors:** Badir Delf Loveymi, Mitra Jelvehgari, Parvin Zakeri-Milani, Hadi Valizadeh

**Affiliations:** a ***Faculty of Pharmacy, Tabriz University of Medical Sciences, Tabriz, Iran.***; b ***Biotechnology Research Center, Tabriz University of Medical Sciences, Tabriz, Iran.***; c ***Drug Applied Research Center, Tabriz University of Medical Sciences, Tabriz, Iran.***; d ***Liver and Gastrointestinal Diseases Research Center, Tabriz University of Medical Sciences, Tabriz, Iran.***; e ***Research Center for Pharmaceutical Nanotechnology, Tabriz University of Medical Sciences, Tabriz, Iran. ***

**Keywords:** Vncomycin, Nanoparticles, Eudragit RS100, Optimization, Response surface methodology

## Abstract

A Box-Behnken design with three replicates was used for preparation and evaluation of Eudragit vancomycin (VCM) nanoparticles prepared by double emulsion. The purpose of this work was to optimize VCM nanoparticles to improve the physicochemical properties. Nanoparticles were formed by using W1/O/W2 double-emulsion solvent evaporation method using Eudragit RS as a retardant material. Full factorial design was employed to study the effect of independent variables, RPM (X1), amount of emulsifier (X2), stirring rate (X3), volume of organic phase (X4) and volume of aqueous phase (X5), on the dependent variables as production yield, encapsulation efficiency and particle size. The optimum condition for VCM nanoparticles preparation was 1:2 drug to polymer ratio, 0.2 (%w/w) amount of emulsifier , 25 mL (volume of organic phase), 25 mL (volume of aqueous phase), 3 min (time of stirring) and 26000 RPM. RPM and emulsifier concentrations were the effective factors on the drug loading (R2 = 90.82). The highest entrapment efficiency was obtained when the ratio of drug to polymer was 1:3. Zeta (ζ) potential of the nanoparticles was fairly positive in molecular level. In vitro release study showed two phases: an initial burst for 0.5 h followed by a very slow release pattern during a period of 24 h. The release of VCM was influenced by the drug to polymer ratio and particle size and was found to be diffusion controlled. The best-fit release kinetic was achieved with Peppas model. In conclusion, the VCM nanoparticle preparations showed optimize formulation, which can be useful for oral administrations

## Introduction

Vancomycin (VCM) is a glycopeptide antibiotic that inhibits bacterial cell wall synthesis at an earlier stage than the beta-lactam antibiotic. Since the oral absorption of VCM is minimal, it is usually given IV (1). VCM is used for the treatment of infections caused by methicillin-resistant staphylococci. It has a high molecular weight and is water-soluble and poorly absorbable from the gastrointestinal tract (2). The oral absorption of highly polar and macromolecular drugs is frequently limited by poor intestinal wall permeability. Some physicochemical properties that have been associated with poor membrane permeability are low octanol/aqueous partitioning, the presence of strongly charged functional groups, high molecular weight, a substantial number of hydrogen-bonding functional groups and high polar surface area (3, 4).

Many therapeutic compounds such as antibiotics and peptide and protein drugs require the use of some kind of absorption enhancer to obtain reasonable plasma concentrations. By loading antibiotics into the nanoparticles, one can expect improved delivery to infected cells. Nanoparticles are the carriers developed for these logistic targeting strategies and are colloidal in nature, biodegradable and similar in behavior to intracellular pathogens. These colloidal carriers, when administered intravenously, are rapidly taken up by the cells of the mononuclear phagocyte system, the very cells which may constitute a sanctuary for intracellular bacteria (5, 6). Therefore, the entrapment of antibiotics within nanoparticles has been proposed for the treatment of intracellular infections (5). The encapsulation of VCM in liposomes and microspheres has been described in previous works (1-3). It has been proposed that VCM-PLGA-loaded microspheres may show a better bioavailability than the free drug (3). Eudragit RS 100 is a polymer commonly used for the preparation of controlled-release oral pharmaceutical dosage forms. Eudragit RS100 contains different amounts of quaternary ammonium groups ranging from 4.5-6.8% and is a neutral copolymer of poly (chlorotrimethyl-ammonioethyl methacrylate). As Eudragit RS 100 is insoluble at physiological pH values, it has been used as a good polymer for the preparation of pH-independent sustained-release formulations of drugs (7). Various non-biodegradable polymers with good biocompatibility such as Eudragit and ethyl cellulose have been used in the preparation of microspheres. Polymethylmethacrylate microspheres were extensively used as bone cement materials in antibiotic releasing agents for bone infection and bone tumors (7). Double-emulsion solvent extraction/evaporation technique is the most commonly used method to encapsulate hydrophilic drugs, especially protein and glycoprotein drugs, into polymeric microspheres (8). Indeed, the presence of a polymeric wall provides a protection from the gastrointestinal environment and may favor a prolonged contact with the epithelium that may be sufficient to increase the bioavailability of certain drugs.

Response surface methodology is a useful tool in the development and optimization of controlled release nanoparticles (9). Different steps involved in response surface methodology include experimental design, regression analysis, constraint optimization and validation.

Response surface methodology (RSM) is a widely practiced approach in the development and optimization of drug delivery devices. Based on the principle of design of experiments (DoEs), the methodology encompasses the use of various types of experimental designs, generation of polymonal equations, and mapping of the response over the experimental domain to determine the optimum formulation(s) (10). The technique requires minimum experimentation and time, thus proving to be far more effective and cost-effective than the conventional methods of formulating dosage forms.

In the present investigation, the effect of factors (rpm, volume of organic phase, aqueous phase, time of stirring and concentration emulsifier) that can influence the drug loading, loading efficiency, particle size and production yield of VCM nanoparticles from Eudragit RS was investigated.

## Experimental

Vancomycin hydrochloride was obtained from Jaberabne Hyan Pharmaceutical Company, Iran. Edragit RS 100 was obtained from RÖhm Pharma GMBh (RÖhm Pharma GMBh, Weiterstadt, Germany), Poly vinyl alcohol (PVA) with molecular weight of MW 95000 was obtained from Acros Organics (Acros Organics, Geel, Belgium) and dichloromethane, methanol, glacial acetic acid, triethanolamine, hydrochloric acid, potassium chloride, sodium chloride, sodium hydrogen phosphate (dibasic), and potassium dihydrogen phosphate were obtained from Merck (Merck, Darmstadt, Germany). Silastic membrane (#10,000 Da) was provided by Biogene (Mashhad, Iran). All other materials used were of analytical or HPLC grade. 

*Experimental design*

The experimental design was a modified Box-Behnken design for five variables. This design was suitable for exploring quadratic response surfaces and constructing second-order polynomial models. Four independent formulation variables analyzed during the study including the amounts of emulsifier (X1), volume of organic solvent (X2), and the amount of dispersing medium (X3), time of stirring (X4) and rate of stirring (X5). The investigated dependent variables were the drug content (DC, Y1), loading efficiency (LE, Y2), particle size (PS, Y3), and production yield (PY, Y4). The complete design consisted of 27 experimental points, which are included three replications. The 81 experiments were carried out in random order. Data were analyzed to fit the polynomial equation to Y (9).

*Preparation of nanoparticles*

VCM-loaded Eudragit RS100 nanoparticles were prepared by W1/O/W2 solvent evaporation method using different ratios of drug to polymer (1:1, 1: 2 and 1: 3). Briefly, 5 mL of aqueous internal phase (containing 100 mg VCM) was emulsified for 15 sec in 20 mL of methylene chloride (containing 100, 200 and 300 mg Eudragit RS100) using homogenizer (22000 rpm). This primary emulsion was poured into 25 mL of a 0.2% PVA aqueous solution while stirring using a homogenizer for 3 min, immersed in an ice water bath, to create the water in oil-in-water emulsion. Three to four mL of NP suspension was obtained after the solvent evaporation under reduced pressure (Evaporator, Heidolph, USA). Nanoparticles were separated from the bulk suspension by centrifugation (Hettich universal 320R, USA) at 22,000 *g *for 20 min. The supernatant was kept for drug assay as described later and the sediment nanoparticles were collected and washed with three portions of 30 mL water and were redispersed in 5 mL of purified water before freeze-drying. Blank nanoparticles (without drug) were prepared under the same conditions (11, 12).

*Micromeritic properties*

A laser light scattering particle size analyzer (SALD-2101, Shimadzu, Japan) was used to determine the particle size of the drug, polymer and nanoparticulate formulations. Samples were suspended in distilled water (nanoparticles and polymer) or acetone (drug) in a 1 cm cuvette and stirred continuously during the particle size analysis.

*Zeta potential measurement*

Zeta (*ζ*) potential measurements of diluted samples were made with a ZetaSizer (Malvern Instruments Ltd., Malvern, UK). Zeta potential values obtained from ZetaSizer were average values from twenty measurements made on the same sample. Initial measurements on several samples of the same kind showed that this number is sufficient to give a representative average value. VCM nanoparticles were diluted with deionized water before the measurement.

*Loading efficiency and production yield (%) determination*

The drug concentration in polymeric particles was determined spectrophotometrically (UV-160, Shimadzu, Japan) at 280.2 nm by measuring the amount of non-entrapped VCM in the external aqueous solution (indirect method) before freeze-drying. In the case of nanoparticles, the external aqueous solution was obtained after the centrifugation of colloidal suspension for 20 min at 22,000 g.

The loading efficiency (%) was calculated according to the following equation: 

Loading efficiency(%) = (actual drug content in nanoparticles/theoretical drug content) × 100 

The production yield of the nanoparticles was determined by accurately calculating the initial weight of the raw materials and the last weight of the polymeric particles obtained. All of the experiments were performed in triplicate (Table 1). 

**Table 1:** Effect of drug: polymer ratio on drug loading efficiency, production yield, particle size zeta potential and polydispersity index of vancomycin nanoparticles

**Formulation code**	**Drug: polymer** **ratios**	**Production yield** **(% ± SD)**	**Theoretical drug** **Content (%)**	**Mean drug Entrapped** **(% ± SD)**	**Drug loading efficiency** **(% ± SD)**	**Mean particle Size** **(nm±SD)**	**Zeta Potential** **(mV±SD)**	Polydispersity Index (PDI)
F1	1:1	96.38 ± 1.65	50	30.25 ± 1.04	63 ± 2.19	362 ± 29.26	18.1±8.82	0.0099
F2	1:2	97.84 ± 1.54	33.33	29.79 ± 1.12	89.37 ± 2.36	430 ± 31.94	25.7±9.72	0.0055
F3	1:3	98.35 ± 1.87	25	23.69 ± 1.02	94.76 ± 1.95	499 ± 110	24.1±7.17	0.0034

*In-vitro release study*

VCM dissolution patterns from freeze-dried nanoparticles were obtained under sinking conditions. Dissolution studies were carried out using a dialysis bag rotating method. A set amount of nanoparticles (20 mg of drug) was added to 200 mL dissolution medium (phosphate buffered saline, pH = 7.4), preheated and maintained at 37 ± 1°C in a water bath, then stirred at 100 rpm. Then, 3 mL of solution was withdrawn at appropriate intervals (0.5, 1, 2, 3, 4, 5, 6, 8, 12 and 24 h). The filtrate (VCM) was replaced by 3 mL of fresh buffer. The amount of VCM in the release medium was determined by UV at 279.8 nm (12, 13). 

In order to have a better comparison between different formulations dissolution efficiency (DE), t50% (dissolution time for 50% fraction of drug) and difference factor, f1 (used to compare multipoint dissolution profiles) were calculated and the results are listed in Table 2.

**Table 2:** Comparison of various release characteristics of vancomycin from different nanoparticle formulations and physical mixture

Difference Factor (f1)	dQ24	cQ0.5	bDE	at 50% (h)	Formulation
38.11	95.03±2.17	11.44±0.99	81.44	3.25	F1
40.52	88.27±0.77	12.27±1.25	76.25	2.24	F2
52.55	82.23±0.78	9.95±0.49	66.37	4.85	F3
0.04	98.70±0.52	96.10±0.43	98.03	0.26	Physical mixture

 DE is defined as the area under the dissolution curve up to a certain time (*t*), expressed as a percentage of the area of the rectangle arising from 100% dissolution in the same time. The areas under the curve (AUC) were calculated for each dissolution profile by the trapezoidal rule (14). DE can be calculated by the following: 

DE **= **t dt 100 ∫ y 

Here, *y *is the drug percentage dissolved at time *t*. All dissolution efficiencies were obtained with *t *equal to 1440 min. The *in-vitro *release profiles of different nanoparticle formulations were compared with physical mixture formulation using difference factor (f1), as defined by: 

f1= (Σ t = 1n |Rt - Tt|) / (Σ t = 1n Rt) × 100 

Here, *n *is the number of time points at which %dissolved was determined. *Rt *is the %dissolved of one formulation at a given time point and *Tt *is the %dissolved of the formulation to be compared at the same time point. The difference factor fits the result between 0 and 15, when the test and reference profiles are identical and approaches above 15 as the dissimilarity increases. 

Data obtained from *in-vitro *release studies were fitted to various kinetic equations to find out the mechanism of drug release from the Eudragit RS100 nanoparticles. The kinetic models used were: 

Qt = k0t (zero-order equation) 

ln Qt = ln Q0 – k1.t (first-order equation) 

Qt = K. S. t0.5= kH. t0.5 

(Higuchi equation based on Fickian diffusion) 

Here, Q is the amount of drug release in time t, Q0 is the initial amount of drug in the nanoparticles, S is the surface area of the nanoparticle and k0, k1 and kH are rate constants of zero order, first order and Higuchi equation, respectively. In addition to these basic release models, the release data was fitted to the Peppas and Korsmeyer equation (power law): 

Mt/M∞ = k.tn 

Here, Mt is the amount of drug release at time t and M∞ is the amount release at time t = ∞, thus Mt/M∞ is the fraction of drug released at time t, k is the kinetic constant, and *n *is the diffusion exponent which can be used to characterize the mechanism of drug release (14, 15).

*Optimization of the VCM nanoparticles*

Response surface methodology (RSM) is a very useful statistical technique for the optimization of VCM formulations. In this design, 5 factors were evaluated, each at 4 levels, and experimental trials were performed at all 27 possible combinations. The amounts of emulsifier (X1), volume of organic solvent (X2) and the amount of dispersing medium (X3), were selected as independent variables. The drug content (DC), loading efficiency (LE), particle size (PS), and percentage production yield (PY) were dependent variables (Table 3).

**Table 3:** Response surface regression Evaluation of VCM formulations in full factorial design

**Formulation code**	**Variable levels in coded form** **†**					**PS (nm)**	**LE (%)**	**DE (%)**	**PY (%)**
	**X1**	**X2**	**X3**	**X4**	**X5**				
F1	26000	15	20	1.5	0.1	480	80	26	94
F2	26000	15	25	3	0.2	502	82	29	95
F3	26000	15	30	4.5	0.4	570	81	28.5	96
F4	26000	20	20	1.5	0.1	490	82	29	97
F5	26000	20	25	3	0.2	468	79	31.2	98.6
F6	26000	20	30	4.5	0.4	510	85	30.2	98.2
F7	26000	25	20	1.5	0.1	580	84	30.8	96.5
F8	26000	25	25	3	0.2	520	88	31.5	98.1
F9	26000	25	30	4.5	0.4	590	88	30.7	98.2
F10	24000	15	20	1.5	0.1	490	83	26.3	94
F11	24000	15	25	3	0.2	476	81	27	95.2
F12	24000	15	30	4.5	0.4	520	86	28.1	95.7
F13	24000	20	20	1.5	0.1	480	77	25.7	94.5
F14	24000	20	25	3	0.2	442	87	28.8	97.7
F15	24000	20	30	4.5	0.4	526	90	30.1	98.1
F16	24000	25	20	1.5	0.1	490	88	29	96.8
F17	24000	25	25	3	0.2	575	90	30.1	97.9
F18	24000	25	30	4.5	0.4	520	90.1	30.3	97.8
F19	22000	15	20	1.5	0.1	450	87.1	25.2	93.4
F20	22000	15	25	3	0.2	440	88	26.5	64.5
F21	22000	15	30	4.5	0.4	473	89	25.8	94.7
F22	22000	20	20	1.5	0.1	410	89	26.3	94.1
F23	22000	20	25	3	0.2	415	93	26.3	96.6
F24	22000	20	30	4.5	0.4	435	91.5	28.2	94.3
F25	22000	25	20	1.5	0.1	465	91.5	27.8	94.1
F26	22000	25	25	3	0.2	472	92.8	29.1	95.3
F27	22000	25	30	4.5	0.4	495	93.1	29.3	95.9

All formulations contained 100 mg VCM, 5 mL water, 200 mg Eudragit RS, 20 mL dichloromethane and 25 mL 0.2% PVA. †X1 is RPM and; X2 is volume of organic solvent,; X3 is dispersing medium; X4 is time of stirring; X5 is concentration of emulsifier; DE indicates drug entrapped; LE, loading efficiency; PS= particle size, PY, production yield.

Various batches of the selected formulation (F2) were made, but the stirring rate was the only parameter that was varied between 22000, 24000 and 26000 rpm. In addition, while keeping the other parameter constant, time of homogenizer stirring was changed (1.5, 3 and 4.5 min). After drying, the weighed batch of nanoparticles was subjected to drug content, loading efficiency, particle size and drug release experiments.

The influence of process variables on nanoparticle formation, micromeritics and drug release characteristics, was investigated. These variables included the emulsifier concentration (0.1, 0.2 and 0.4%) and volume of organic solvent (15, 20 and 25 mL) and dispersing medium (15, 25 and 35 mL).

*Regression analysis*

The targeted response parameters were statistically analyzed by applying one-way ANOVA at 0.05 levels. Individual response parameters were evaluated using the F-test and quadratic models of the form given below were generated for each response parameter using the multiple linear regression analysis (17).

Y = b0 + b1X1+ b2 X2 + b3 X3 + b4 X4 + b5X5 + b11 X12 + b22 X22 + b33 X32 + b44 X42 + b55 X52 + b12 X1 X2 + b13 X1 X3 + b14 X1 X4 + b15 X1 X5 + b23 X2 X3 + b24 X2 X4 + b25 X2 X5 + b34 X3 X4 + b35 X3 X5 + b45 X4 X5

In this equation, Y is the predicted response, X1, X2, X3, X4 and X5 are independent variables, b0 is the intercept, b1, b2, b3, b4 and b5 are linear effects, b12, b13, b14, b15, b23, b24, b25, b34 and b45 are interaction terms. The main effects (X1, X2, X3, X4 and X5) represent the average result of changing one factor at a time from its low to high value. The interaction terms (X1X2, X1X3, X1X4, and X1X5) show how the response changes when five factors are simultaneously changed. The polynomial terms (X1X1, X2X2, X3X3, X4X4 and X5X5) are included to investigate nonlinearity. Three-dimensional surface (3D) plots were drawn to illustrate the main and interactive effects of the independent variables on production yield, drug content, loading efficiency and particle size. The optimum values of the selected variables were obtained from the software and also from the response surface plots.

Numerical optimization using the desirability approach was employed to locate the optimal settings of the formulation variables to obtain the desired response (17). An optimized formulation was developed by setting the constraints on the dependent and independent variables. The formulation developed was evaluated for the responses and the experimental values obtained were compared with those predicted by the mathematical models generated.

## Results and Discussion

A W/O/W multiple emulsion solvent evaporation/extraction method is mostly used for the encapsulation of water-soluble drug and therefore was the method of choice for the water-soluble VCM drug. Droplets of the polymer in organic solution were added to a solution PVA (as stabilizer) aqueous solution. At the end, the uniform-sized beads were collected (18). In the nanoparticles prepared by the evaporation method, the amount of drug entrapped in microspheres was lower than the theoretical value. This indicates that some free drug crystals were lost in the process of encapsulation. By the increase of drug to polymer ratio, the amount of free drug lost is decreased (Table 1) so that at the ratio of 1:3 in drug to polymer, the amount of drug entrapment was 23.69% which was very close to the theoretical value (25%).

The encapsulation efficiency of the drug depends on the solubility of the drug in the solvent and continuous phase (19). Important prerequisites for high encapsulation efficiencies by the W/O/W method are: (1) the insolubility of the drug in the external phase, and (2) the fine dispersion of the aqueous drug solution into the organic polymer solution to form a W/O emulsion (20). VCM is insoluble in methylene chloride used to dissolve the polymer and thus cannot part from the internal into the external phase. Entrapment efficiency of polypeptides is increased by enhancing the viscosity builders (21). Despite the hydrosolubility of VCM, favoring the leakage of the drug into the external aqueous phase, the entrapment efficiencies were rather high (22). It is assumed that VCM is localized at the interfaces (either internal water in oil or external oil in water). Therefore, a significant amount of drug is supposed to be adsorbed at the outer surface. In addition, the removal of the organic solvent under reduced pressure favors its fast evaporation followed by the polymer precipitation and thus, reduces the migration of the drug to the external phase. Indeed, the faster the solvent evaporation, the higher the encapsulation efficiency (22). One possible explanation could concern the increase of the primary emulsion viscosity due to the different VCM concentrations studied which could reduce the leakage of the drug towards the external aqueous phase (23). Generally, increasing the polymer to drug ratio increased the production yield, when the ratio of polymer-drug increased from 1:1 to 1:3, the production yield was increased (p > 0.05). Size of microspheres was found to be increased with the increase in the concentration of polymer (Table 1). It can be attributed to the fact that with the higher diffusion rate of non-solvent to polymer solution, the smaller size of microcapsules is easily obtained (22, 23). A volume-based size distribution of drug, polymer, and drug-loaded nanoparticles indicated a log-probability distribution. Mean particle size of F3 was 499 ± 110 nm. The data describing the particle sizes of the nanoparticles are given in Table 1. As it can be seen, the particle size is increased with increasing the polymer amount. It has already been reported that particle size is proportional to the viscosity of dispersed phase (10, 23-25). In fact, viscosity of dispersed phase was increased from F1 (1:1) to F3 (1:3). When the viscosity of the dispersed phase of these formulations was investigated, it was found that particle sizes of nanoparticles were directly proportional to the apparent viscosity of dispersed phase. The results showed that the apparent viscosities of the different drugs polymer ratios (1 : 1, 1 : 2 and 1 : 3) were 13, 16 and 18.8 mPa.S respectively. When the dispersed phase with higher viscosity was poured into the dispersion medium, bigger droplets were formed with larger mean particle size.

The zeta potential of three nanosphere formulations, VCM and Eudragit RS100 are shown in Table 1. Blank nanoparticles had positive charge (15.7 mV). Drug-loaded nanoparticle indicated more positive charge, which could be ascribed to the cationic nature of VCM. Zeta potential is the potential difference, the dispersion medium and the stationary layer of fluid attached to the dispersed particle. A value of potential zeta (positive) can be taken as the arbitrary value that separates low-charged surfaces from highly-charged surfaces. The significance of zeta potential is that its value can be related to the stability of colloidal dispersion. The zeta potential indicates the degree of repulsion between adjacent, similarly charged particles in dispersion. For molecules and particles that are small enough, a high zeta potential will confer the stability, *i.e. *the solution or dispersion will resist aggregation (23, 27).

The *in-vitro *release profiles of VCM from nanoparticles (Table 2) exhibited initial burst effect, which may be due to the presence of some drug particles on the surface of the nanoparticles. In most cases, a biphasic dissolution profile was observed at pH of 7.4 as follows: the initial rapid drug leakage generally ended very early and for the remaining time, nearly linear behavior was observed. The first portion of the dissolution curves is due to VCM dissolution, which starts immediately after the beginning of the test for the portion of drug on the surface of nanoparticles. After such a phase, two phenomena can be combined in enhancing in the diffusion of the remaining dispersed drug into the bulk phase as well as the formation of pores within the matrix due to the initial drug dissolution; particle wetting and swelling which enhances the permeability of the polymer to the drug (Table 2). The results indicated that some factors such as drug-polymer ratio governed the drug release from these nanoparticles. Drug release rates were decreased with increasing the amounts of polymer in the formulation (Table 2). Higher level of VCM corresponding to lower level of the polymer in the formulation resulted in an increase in the drug release rate (F1). As more drugs are released from the nanoparticles, more channels are probably produced, contributing to faster drug release rates. However, Table 2 shows that the burst effect is lower when the drug to polymer ratio is 1:3 (F3) compared with other formulations. In the formulation F3, a decreased diffusivity due to the high polymer concentration could reduce the leakage of the drug towards the dissolution medium and decrease the burst effect (to compare with F1 and F2). VCM nanoparticles of each formulation displayed an immediate and important initial drug release in the first 2 h (22-40%), followed by an 84-87% during 24 h (Table 2). An immediate high release may be due to the small diameter of nanoparticles leading to a large exchange surface and probably to a more porous structure owing to the solvent evaporation method, favoring the release of the encapsulated drug (28). Indeed, it has been already demonstrated that the slow precipitation of nanoparticles after the solvent evaporation leads to more porous particles compared to the fast polymer precipitation obtained after the solvent extraction (23). The presence of Eudragit RS100 in the matrix of nanoparticles conferred a slower and more progressive release of VCM during the time of the experiment (11). Therefore, any mechanism which is able to restrict the diffusion of VCM towards water would be easily observed, due to the slow diffusion of water into the lipophilic Eudragit RS100 matrix (7, 11, 23-25). F1, F2 and F3 nanoparticles showed lower dissolution efficiency, *i.e. *slower dissolution in comparison with respective physical mixture (p < 0.05), (Table 2). According to Table 2, the lowest DE was observed for F3 (66.37%) and the dissolution efficiency of the physical mixture was 98.03% (p < 0.05). The value of t50% varied between 2.24 (F2 formulation) and 4.85 h (F3 formulation). The results of similarity factor (f2) showed that the release profile of nanoparticle formulations is different from that of physical mixture (Table 2).

The independent variables and their levels were selected based on the preliminary trials undertaken. The %DT, %LE, PS and %PY for the formulations (F1 to F27) showed a wide variation. The data clearly indicated that the %DT, %LE, PS and %PY values are strongly dependent on the selected independent variables. The fitted equations (full and reduced) that relates the responses PY and %F to the transformed factors are shown in Table 4. 

**Table 4:** Equations Response surface regression

**Equation of regression coefficients**	**Term**
PY = 628.18 + 0.01 X1 - 1.70 X2 - 64.27 X3 + 85.17 X4 + 2134.91 X5 - 0.08 X22 - 14.14 X44 - 0.04 X1X5 + 0.70 X2X3 - 23.49 X2X5	PY versus X1, X2, X3, X4, X5
DE= 145.828 - 0.003 X1 - 0.374 X2 - 9.184 X3 + 30.317 X4 + 276.106 X5 + 0.005 X22 - 4.269 X44 - 0.004 X1X5 + 0.006 X2X3 - 0.349 X2X5	DE versus X1, X2, X3, X4, X5
LE = 231.175 - 0.014 X1 -3.443 X2 + 4.318 X3+ 3.412 X4 + 65.562 X5 + 0.041 X22 - 4.469 X44 + 0.009 X1X5 + 0.128 X2X3 - 4.59 X2X5	LE versus X1, X2, X3, X4, X5
PS = -295.585 + 0.264 X1 -103.5 X2 -86.137 X3 +620.407 X4 -197.217 X5 + 1.746 X22 - 47.818 X44 - 0.005 X1X3 + 0.163 X1X5 + 1.685 X2X3 - 56.882 X2X5	PS versus X1, X2, X3, X4, X5

The polynomial equations can be used to draw conclusions after considering the magnitude of coefficient and the mathematical sign it carries (*i.e., *positive or negative). Table 4 shows the results of the analysis of variance (ANOVA), which was performed to identify the insignificant factors. The high values of correlation coefficient for %DC, %LE, %PY and PS indicate a good fit *i.e., *good agreement between the dependent and independent variables. The equations may be used to obtain estimates of the response as a small error of variance was noticed in the replicates. The significance test regression coefficient was performed by applying the Student F-test.

The results of statistical analysis are shown in Table 5. The coefficients b1, b2, and b11 were found to be significant at p < 0.05; hence they were retained in the reduced model. 

**Table 5:** Calculations for testing the model in portions.

**Response/coefficients**	**DF**	**SS**	**MS**	**F** **(cal)**	**R2**	**p**	**MPE (%)**
PY	13	639.58	49.20	1.65	62.24	0.190	6.54
DE	13	84.48	6.50	9.89	90.82	0.000	2.14
LE	13	456.246	35.10	5.09	83.59	0.003	2.60
PS	13	52135.5	4010.42	6.84	87.25	0.001	2.77

**Figure 1:**
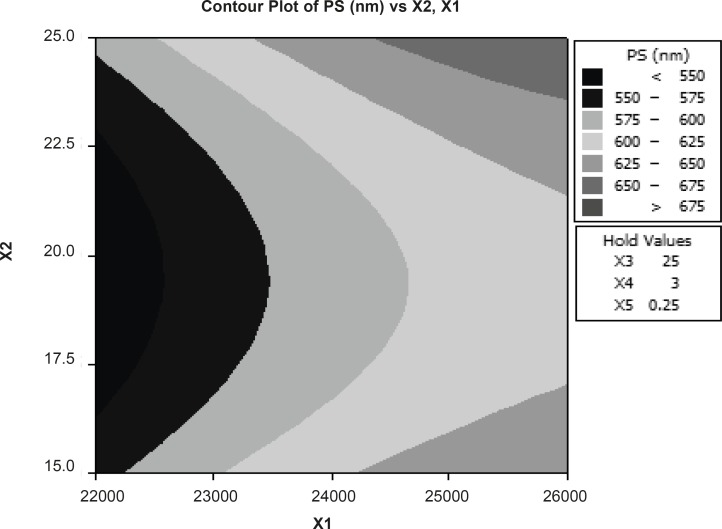
Response contour plot showing the effect of formulation variables (X1=RPM and X2= volume of organic phase) on percent particle size (X3=volume of organic phase, X4=volume of aqueous phase and X5= concentration emulsifier were constant).

The reduced model was tested in portions to determine whether the coefficients b12 and b22 contribute significant information for the prediction of PY or not. The results for testing the model in portions are. The results of multiple linear regression analysis (response surface regression) reveal that, on increasing the amount of emulsifier and volume of aqueous phase, increased DE is observed; the coefficients b1 and b3 bear a negative sign. The results of statistical analysis are shown in Table 4. The reduced model was tested in portions to determine whether the coefficients b11, b22, and b12 contribute significant information for the prediction of %F or not. The results for testing the model in portions are depicted in Table 5. Hence, conclusions can be drawn considering the magnitude of the coefficient and the mathematical sign (positive or negative) it carries. According to Figure 1, particle size is dependent on major independent factors such as rpm and volume of organic phase. The results show that linear and interaction components in the proposed model are not very significant (R2 = 87.25).

**Figure 2:**
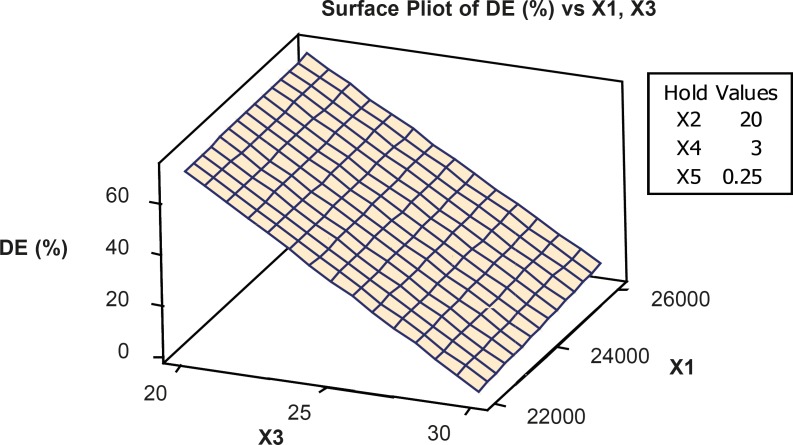
Response surface plot showing the effect of formulation variables (X1=RPM and X3=volume of aqueous phase) on percent drug loading (X2= volume of organic phase, X4=time of stirring and X5=concentration emulsifier were constant).

The optimum condition for VCM nanoparticles preparation was 1:2 (v/w) drug to polymer ratio, 0.2 (%w/w) amount of emulsifier, 25 mL (volume of organic phase), 25 mL (volume of aqueous phase), 3 min (time of stirring) and 26000 rpm. Emulsifier concentrations and rpm were the most effective factors on the drug loading (R2 = 90.82) (Table 5 and Figure 2). 

**Figure 3:**
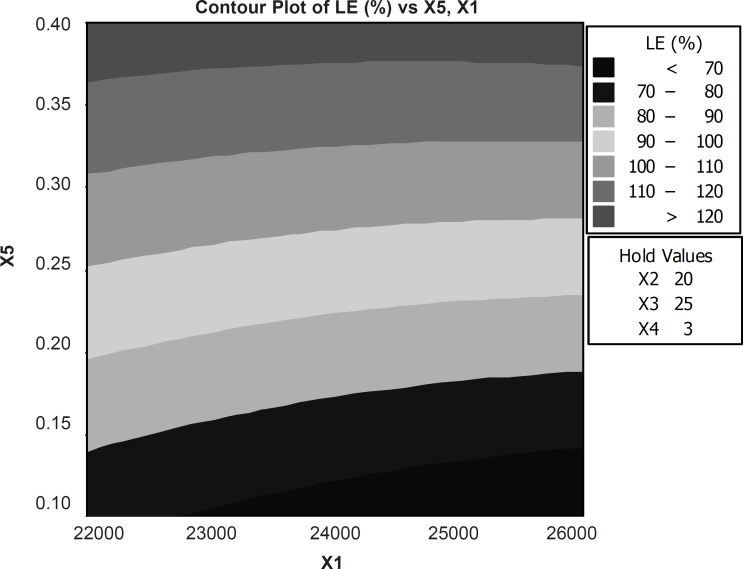
Response contour plot showing the effect of formulation variables (X1=RPM and X5= concentration emulsifier) on percent loading efficiency (X2= volume of organic phase, X3=volume of aqueous phase and X4=time of stirring were constant).

The results obtained from the predicted model were used to create a contour plot for loading efficiency and the production yield is shown in Figure 3. Emulsifier concentration and rpm affect on the loading efficiency and production yield (R2 = 83.59).

An increase in the concentration of VCM leads to an increase in drug loading and loading efficiency since the coefficient b2 bears a positive sign. An increase in the time of stirring leads rpm and concentration of emulsifier to an increase in the mean particle size as the coefficient b2 bears a positive sign. The increase in rpm results in decreased PS values (Table 5).

The *in-vitro *release profiles were fitted on various kinetic models in order to find out the mechanism of drug release (29, 30). The rate constants were calculated from the slope of the respective plots. High correlation was observed for the Peppas model. The data obtained were also put in Korsmeyer-Peppas model in order to find out n-value, which describes the drug release mechanism. The n-value of microspheres of different drug to polymer ratio was in the range of 0.51-0.91 (Table 6), indicating that the mechanism of the drug release was diffusion and erosion controlled. 

**Table 6:** Fitting parameters of the *in-vitro *release data to various release kinetic models for nanoparticles.

**Order**		**Physical Mixture**	**F3**	**F2**	**F1**
Zero f=kt	K0	0.0358	0.0337	0.0339	0.0130
	RSQ	0.6134	0.5425	0.6859	0.0123
	MPE %	31194926.04	30467176.57	2054809.332	7483066.126
First Ln (1-f)=kt	K1	-0.1363	-0.0939	0.0770	-0.2810
	RSQ	0.8280	0.7145	0.8280	0.6887
	MPE %	28090095.32	33443245.23	1926126.997	8798802.138
Peppas Lnf=lnk+blnt	b	0.38	0.42	0.47	0.0072
	kp	0.2176	0.2120	0.1990	0.1157
	RSQ	0.8672	0.8187	0.8341	0.2773
	MPE %	20.0090	26.0783	22.1364	11.4726
Higuchi f=kt 0.5	Kh	1.4696	1.5256	1.5955	0.0072
	RSQ	0.5093	0.5320	0.4955	0.5093
	MPE %	58426092	6334302.63	3346803.339	98283051.38

## Conclusion

VCM nanoparticles were prepared using double emulsion (W1/O/W2) solvent evaporation method. Drug polymer ratio, stirring speed, time of stirring, emulsifier, dispersing medium and organic solvent influenced the characteristics of the nanoparticles. The entrapment efficiency was high for all formulations. It was observed that at higher drug concentration, the mean particle size of the nanoparticles is high but increasing the stirring speed and emulsifier content, resulted in smaller mean particle size of nanoparticles. High correlation was observed for the Peppas model. The data obtained were also put in Korsmeyer-Peppas model in order to find out n-value, which describes the drug release mechanism. The n-value of nanoparticles of different drug to polymer ratio was in the range of 0 < n < 0.5, indicating that the mechanism of the drug release was diffusion controlled. It was suggested that the mechanism of drug release from nanoparticles was diffusion-controlled. A response surface methodology has been employed to produce VCM nanoparticles for oral drug delivery in Eudragit RS by double emulsion. The formulation variables studied exerted a significant influence on PY, DC, LE and PS. The obtained results indicate that the response surface methodology can be employed successfully to qualify the effect of several formulation and processing variables and thereby, minimize the number of experimental trials and reduce the formulation development cost.
